# Lithium carbonate bridging and outcomes of radioiodine therapy in severe Graves’ disease: a retrospective cohort study

**DOI:** 10.3389/fendo.2026.1770772

**Published:** 2026-05-07

**Authors:** Xuemei Gao, Wei Chang, Ke Sun, Baoping Liu, Qiao Ruan, Xingmin Han, Ruihua Wang

**Affiliations:** Department of Nuclear Medicine, The First Affiliated Hospital of Zhengzhou University, Zhengzhou, China

**Keywords:** bridging therapy, Graves’ disease, lithium carbonate, radioiodine therapy, thyroid storm

## Abstract

**Objective:**

This study evaluated whether a short−term lithium carbonate bridging therapy was associated with improved outcomes of subsequent radioiodine (RAI) therapy in patients with severe Graves’ disease during the high−risk interval after antithyroid drug (ATD) cessation.

**Methods:**

In this single−center retrospective cohort, adults with severe Graves’ disease undergoing RAI were included. The lithium group (n=46) received lithium (serum target 0.6–1.0 mmol/L) from ATD withdrawal until 7 days post−RAI, while the standard−care group (n=100) received routine management without lithium bridging. The primary outcome was thyroid function status at 6 months, defined biochemically: euthyroidism (normal TSH, FT3, FT4 within reference ranges), hypothyroidism (elevated TSH with low FT4), or treatment failure (persistent hyperthyroidism or need for repeat RAI/surgery within 6 months).

**Results:**

Lithium bridging was associated with a higher euthyroidism rate (30.4% vs. 13.0%, *P* = 0.009) and a lower hypothyroidism rate (54.3% vs. 77.0%, *P* = 0.005) compared to standard care. This was preceded by a significant reduction in serum FT3 and FT4 following lithium initiation (P < 0.001). Treatment was well tolerated, with mild gastrointestinal side effects in 8.7% and no cases of thyroid storm (0% vs. 3.0% in standard−care group). Multivariate analysis confirmed pre−RAI FT3 as a key modifiable risk factor for treatment failure (adjusted OR 1.55 per 5 pmol/L increase, 95% CI 1.15–2.09, *P* = 0.004). Within the lithium group, the magnitude of FT3 reduction (ΔFT3) was independently associated with treatment success (adjusted OR 0.45 per 5 pmol/L decrease, 95% CI 0.22–0.92, *P* = 0.028).

**Conclusion:**

Pre−RAI lithium carbonate bridging may be associated with improved therapeutic outcomes in severe Graves’ disease, potentially mediated through reduction in serum FT3. These findings support the need for prospective randomized studies to confirm the role of lithium as a bridging strategy in high−risk patients.

## 1 Introduction

Radioiodine (RAI) therapy is a definitive treatment for Graves’ disease (1, 2). However, its efficacy can be suboptimal, and its administration necessitates an antithyroid drug (ATD)-free period, creating a high-risk window for rebound thyrotoxicosis and life-threatening complications such as thyroid storm, especially in severe cases (3, 4). This presents a dual challenge: mitigating acute peri−procedural risk and improving long-term treatment success. Current management of this interval is variable and often passive, relying primarily on symptom control with beta-blockers (5), highlighting an unmet need for strategies that actively optimize the conditions for RAI therapy.

The success of RAI itself is not guaranteed. A higher pre-ablation thyroid hormone level, particularly free triiodothyronine (FT3), is a robust predictor of treatment failure, correlating with persistent hyperthyroidism (6, 7). Pathophysiologically, elevated FT3 reflects a hyperactive gland state with accelerated follicular cell turnover and potentially reduced expression of the sodium iodide symporter (NIS), leading to functional radioresistance (8, 9). Therefore, actively reducing FT3 prior to RI is not merely supportive but may represent a direct strategic lever to potentially enhance therapeutic efficacy by modulating a key determinant of radioresponsiveness.

Lithium carbonate, a mood stabilizer, offers a unique pharmacological profile for this purpose. It inhibits thyroid hormone secretion by blocking thyroglobulin proteolysis (10). Its impact on thyroid function has been documented (11), and its short-term use has thus been proposed as a potential “bridge” to maintain hormonal control during the vulnerable pre-RAI period (10). It is hypothesized that this positions lithium as a candidate agent to actively optimize the pre−ablation milieu. However, comprehensive evidence is lacking regarding whether this bridging strategy translates into improved final outcomes, its safety in this specific context, and—most critically—whether its benefit is mechanistically achieved through the reduction of the pivotal risk factor, FT3.

This retrospective cohort study was therefore designed to evaluate the association of lithium carbonate bridging with RAI treatment outcomes in severe Graves’ disease. Specifically, we aimed to: 1) compare outcomes and safety of this strategy versus standard care; 2) identify independent predictors of outcome, with a focus on pre RAI hormone levels; and 3) investigate within the lithium group whether the degree of FT3 reduction quantitatively correlates with therapeutic success, thereby exploring a potential mechanism for optimization.

## 2 Methods

### 2.1 Study design and reporting

This single-center, retrospective cohort study was conducted to evaluate the strategy of lithium carbonate bridging for optimizing radioiodine therapy outcomes in severe Graves’ disease. The study was performed at the First Affiliated Hospital of Zhengzhou University between December 2022 and May 2025. It complies with the Declaration of Helsinki and is reported according to the Strengthening the Reporting of Observational Studies in Epidemiology (STROBE) guideline. The study protocol was approved by the Institutional Review Board (Approval No: 2024-KY-0016), which waived the requirement for informed consent due to the retrospective nature of the analysis.

### 2.2 Participants

Consecutive adult patients (≥18 years) with a definitive diagnosis of Graves’ disease who underwent planned definitive radioiodine therapy for severe hyperthyroidism were identified through electronic medical record search using ICD 10 codes and RAI procedure codes. Severe disease was defined at the time of radioiodine decision (after antithyroid drug washout) by one or more of the following criteria, consistent with features associated with poor RAI response or high risk of thyrotoxicosis related complications as per current guidelines (3, 4):

1. Marked biochemical severity: FT3 >15 pmol/L or FT4 >50 pmol/L.
2. Presence of major complications: Documented atrial fibrillation, heart failure, or thyrotoxic periodic paralysis.
3. Large goiter: Estimated thyroid weight >50g by ultrasound, a factor consistently associated with increased therapeutic challenges (12).
4. ATD intolerance or poor response: Including major side effects (e.g., agranulocytosis, severe hepatitis) or failure to achieve adequate control after a standard course of ATDs, indicating refractory disease necessitating definitive therapy (13).

Exclusion criteria were: Previous thyroid surgery or radioiodine therapy; pregnancy or lactation; severe renal impairment (estimated glomerular filtration rate <30 mL/min/1.73m²); concurrent use of other thyroid-blocking agents; or incomplete clinical or laboratory data required for the 6-month follow-up period.

**Figure 1:**
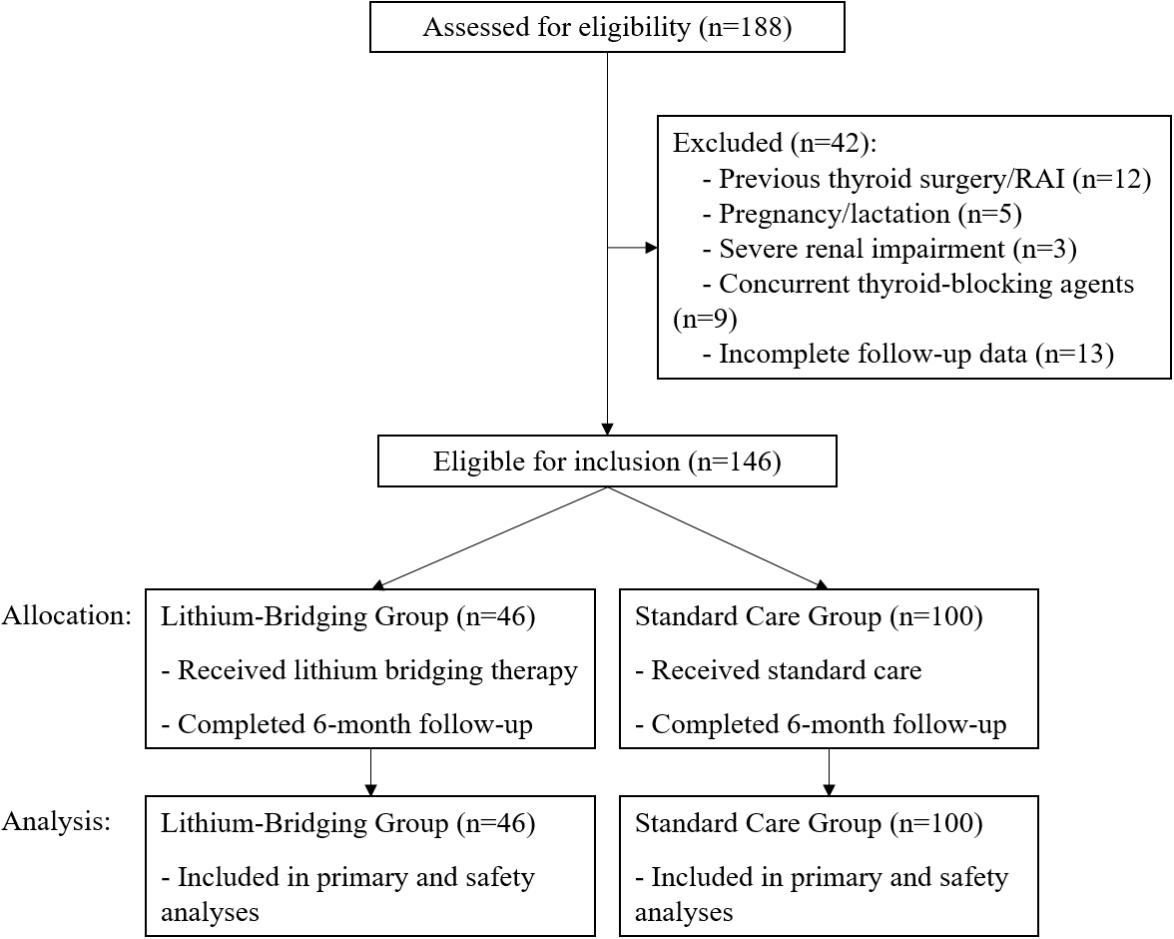
Participant flow diagram. This diagram illustrates the screening, inclusion, and exclusion process for the retrospective cohort study evaluating the strategy of lithium carbonate bridging to optimize radioiodine therapy outcomes in patients with severe Graves’ disease. A total of 186 consecutive patients scheduled for radioiodine therapy were screened between December 2022 and May 2025. Forty patients were excluded based on predefined criteria. The remaining 146 eligible patients were included in the final analysis and categorized into two groups: the Lithium−Bridging Group (n=46), who received lithium carbonate bridging therapy, and the Standard−Care Group (n=100), who received routine preoperative management without lithium. The diagram is presented in accordance with STROBE guidelines.

A total of 186 consecutive patients were screened. After applying exclusion criteria, 146 eligible patients were included in the final analysis: 46 received lithium bridging and 100 received standard care (**Figure 1**).

### 2.3 Institutional treatment context

At our center, RAI is offered to patients with Graves’ disease who have contraindications to or preference against surgery, and those with uncontrolled hyperthyroidism despite ATDs or with ATD intolerance. Lithium bridging is considered in patients with high pre RAI FT3/FT4 levels (above the thresholds described) or large goiter (>50g), with the aim of reducing thyroid hormone levels and theoretically enhancing RAI efficacy. The use of RAI for Graves’ disease has declined regionally in recent years due to concerns regarding thyroid eye disease and delayed therapeutic effect; however, it remains a first line definitive option in patients without active moderate to severe ophthalmopathy.

### 2.4 Thyroid volume and weight estimation

Thyroid weight was estimated from volume measurements obtained via standardized ultrasonography. The volume of each thyroid lobe was calculated using the ellipsoid formula: Volume (mL) = length (cm) × width (cm) × thickness (cm) × π/6. The isthmus volume was calculated separately if it was visibly enlarged (thickness >3 mm) and added to the total. Total thyroid volume (mL) was then converted to estimated weight (grams) by assuming a tissue density of approximately 1 g/mL. All ultrasound examinations were performed by experienced sonographers using a high-frequency linear-array transducer (GE Logiq E9), and the maximum dimensions in three planes were recorded for calculation.

### 2.5 Laboratory measurements and therapeutic monitoring

Serum FT3 and FT4 levels were measured using standardized chemiluminescent immunoassays (Roche Diagnostics, Mannheim, Germany). Free T3 (FT3) is the standard parameter used in our institution for assessment of thyrotoxicosis, consistent with common practice in many centers; total T3 was therefore not measured. The institutional reference ranges were: FT3, 3.28–6.47 pmol/L; FT4, 7.9–18.4 pmol/L. For patients in the lithium group, serum lithium levels were monitored to ensure safety and therapeutic efficacy. Monitoring typically involved checking the serum level 5–7 days after initiation or dose change, and then weekly until RAI administration, with the dose titrated to maintain a target therapeutic range of 0.6–1.0 mmol/L, as recommended for its thyroid effect (10). All monitored patients (100%) achieved and maintained levels within the target range during the bridging period.

### 2.6 Study interventions and groups

Lithium-bridging group: Patients received lithium carbonate orally, initiated on the day of ATD withdrawal. The starting dose was 300–600 mg/day, divided into two or three doses, based on clinician judgment and patient factors (e.g., age, renal function). The dose was titrated based on serum lithium levels to achieve and maintain the target range of 0.6–1.0 mmol/L. Lithium was continued until 7 days after RAI administration, a duration designed to bridge the period of highest risk for hormone rebound and potential thyroid storm, covering both the pre-RAI interval and the initial post-therapy phase when inflammatory hormone release can occur.

Standard-care group: Patients received the institution’s routine preoperative management, which typically involved a planned ATD−free period of several days prior to radioiodine without specific bridging pharmacotherapy, other than beta−blockers for symptomatic control. This group serves as the reference for current standard practice.

All patients, regardless of group, received a standard calculated dose of I-131. The activity was determined by the treating nuclear medicine physician based on a standard protocol incorporating thyroid size (estimated weight), the 24-hour RAI uptake value, and clinical judgment, aiming for an ablative dose.

### 2.7 Variables and data collection

Data were extracted from the hospital’s electronic medical record system using a pre-piloted, standardized case-report form to ensure consistency. Missing data were minimal (<2% for any key variable) and were handled by complete-case analysis for the respective models.

Baseline variables included demographics (age, sex), estimated thyroid weight, disease duration (months), TRAb levels (IU/L), cumulative ATD dose (mg), ATD dose at withdrawal (mg/day), Charlson Comorbidity Index, and the presence of pre RAI comorbid conditions documented in the record. These comorbidities were specifically coded as: ATD allergy or significant side effects; thyrotoxic periodic paralysis; significant drug induced liver injury; leukopenia or agranulocytosis; atrial fibrillation, heart failure, or other thyrotoxic heart disease; and significant electrolyte imbalance (e.g., hypokalaemia).
Laboratory variables: For the lithium group, two key sets of thyroid function tests were collected: the ‘initial’ level (measured within 2 days of ATD withdrawal, marking the start of lithium therapy) and the ‘pre-RAI’ level (measured on the day of or the day before RAI administration). For the standard care group, only the pre-RAI FT3 and FT4 levels were systematically available. The change in hormone levels during the bridging period for the lithium group was calculated as ΔFT3 and ΔFT4 (initial value – pre-RAI value).

Outcome variables:

- Primary efficacy outcome: Thyroid function status assessed at 6 months (± 4 weeks) after RAI administration. In routine clinical practice, thyroid function tests (FT3, FT4, TSH) were performed at approximately 1, 3, and 6 months post-RAI. In this retrospective study, these time points were operationalized by extracting the nearest test results within allowable windows (1 month ±2 weeks, 3 and 6 months ±4 weeks); missing 6-month data (<2%) were handled by complete-case analysis. Status was categorized as:
  - (1) Euthyroidism: Thyroid-stimulating hormone (TSH) and free thyroid hormone levels within the normal institutional reference ranges.
  - (2) Hypothyroidism: Elevated TSH with or without low FT4, requiring initiation of levothyroxine replacement therapy.
  - (3) Treatment failure: Persistent hyperthyroidism (elevated FT3 and/or FT4 with suppressed TSH) or need for repeat RAI or thyroidectomy within 6 months.

- Safety outcomes included: (1) Lithium-related adverse events, actively documented in clinical notes (e.g., nausea, abdominal discomfort, tremor, polyuria). Renal function (serum creatinine, eGFR) was monitored at baseline and before RAI administration. (2) Occurrence of post-RAI thyroid storm, diagnosed according to the Burch-Wartofsky Point Scale (BWPS ≥45) (14, 15).

### 2.8 Statistical analysis

Categorical variables were compared using the Chi-square or Fisher’s exact test. Continuous variables were compared using the independent samples t-test or Mann–Whitney U test, as appropriate. Paired tests were used for within-group comparisons in the lithium-bridging group.

Regression analysis: Binary logistic regression was used to identify independent predictors of treatment failure. Variables with *P* < 0.10 in univariate analyses were entered into multivariate models. Multicollinearity among predictors was assessed using variance inflation factor (VIF), with VIF < 5 considered acceptable. Model fit was evaluated using the Hosmer–Lemeshow goodness of fit test.

- Model A (Full Cohort): Assessed whether assignment to the lithium-bridging strategy was independently associated with a reduced odds of treatment failure, adjusting for age, sex, thyroid weight, large goiter (>50g), pre-RAI FT3, and pre-RAI FT4, disease duration, TRAb, cumulative ATD dose, ATD dose at withdrawal, and Charlson Comorbidity Index.
- Model B (Lithium Subgroup): Investigated the mechanistic hypothesis by testing whether the magnitude of FT3 reduction (ΔFT3) was an independent predictor of success, adjusting for age, sex, thyroid weight, large goiter, and the final pre-radioiodine FT3 level.

Continuous predictors were analyzed per 5 pmol/L increment, a clinically relevant difference based on typical changes between controlled and uncontrolled hyperthyroidism in our practice. Sensitivity analyses using continuous FT3 values yielded consistent results (**Supplementary Table 3**). Results are expressed as adjusted odds ratios (aOR) with 95% confidence intervals (CI). A two-tailed *P*-value <0.05 was considered significant. All analyses were performed using SPSS Statistics version 26.0.

## 3 Results

### 3.1 Participant flow and baseline characteristics

Between December 2022 and May 2025, 186 consecutive patients were screened. After applying exclusion criteria, 146 eligible patients were included in the final analysis: 46 received lithium bridging and 100 received standard care (**Figure 1**). Baseline demographic and clinical characteristics were comparable between the two groups on measured variables (**Table 1**). There were no significant differences in age, sex distribution, estimated thyroid weight, disease duration, TRAb levels, cumulative ATD dose, ATD dose at withdrawal, Charlson Comorbidity Index, or the prevalence of pre-radioiodine comorbidities (all *P* > 0.05).

**Table 1:** Baseline characteristics.

Characteristic	Lithium-bridging group (n=46)	Standard-care group (n=100)	*P*-value
Demographics			
Age, years (mean ± SD)	46.2 ± 12.5	43.8 ± 13.2	0.304
Female, n (%)	31 (67.4)	68 (68.0)	0.944
Thyroid morphology			
Thyroid Weight, g (mean ± SD)	42.3 ± 18.2	45.8 ± 24.1	0.345
Large goiter (>50g), n (%)	12 (26.1)	28 (28.0)	0.811
Disease characteristics			
Disease duration, months (median, IQR)	18 (12–36)	16 (10–32)	0.412
TRAb, IU/L (median, IQR)	8.2 (4.1–15.6)	7.5 (3.8–14.9)	0.556
Cumulative ATD dose, g (mean ± SD)	2.8 ± 1.4	2.5 ± 1.3	0.214
ATD dose at withdrawal, mg/day (mean ± SD)	15.2 ± 6.8	16.1 ± 7.2	0.476
Charlson Comorbidity Index (mean ± SD)	1.2 ± 1.1	1.0 ± 0.9	0.301
Pre-radioiodine comorbidities, n (%)			
ATD allergy/side effects	5 (10.9)	13 (13.0)	0.727
Thyrotoxic periodic paralysis	1 (2.2)	4 (4.0)	1.000
Drug-induced liver injury	12 (26.1)	19 (19.0)	0.330
Leukopenia/Agranulocytosis	15 (32.6)	20 (20.0)	0.097
AF/HF/Thyrotoxic heart disease	2 (4.3)	8 (8.0)	0.511
Significant electrolyte imbalance	2 (4.3)	4 (4.0)	1.000

AF, Atrial Fibrillation; HF, Heart Failure; ATD, antithyroid drug; TRAb, TSH receptor antibody. P-values were derived from independent samples t-test, Mann–Whitney U test, Chi-square test, or Fisher’s exact test, as appropriate.

### 3.2 Primary efficacy and safety outcomes

Lithium bridging was associated with a different thyroid function outcome profile at 6 months. Patients receiving lithium had a higher rate of euthyroidism compared to the standard care group (30.4% vs. 13.0%, *P* = 0.009), and a lower rate of hypothyroidism (54.3% vs. 77.0%, *P* = 0.005). The rate of treatment failure was comparable between the two groups (15.2% vs. 10.0%, *P* = 0.345) (**Table 2**).

**Table 2:** Primary outcome: thyroid function at 6 months post-radioiodine.

Outcome	Lithium-bridging group (n=46)	Standard-care group (n=100)	*P*-value
Euthyroidism, n (%)	14 (30.4)	13 (13.0)	0.009
Hypothyroidism, n (%)	25 (54.3)	77 (77.0)	0.005
Treatment failure, n (%)	7 (15.2)	10 (10.0)	0.345

P-values were derived from the Chi-square test.

### 3.3 Thyroid hormone dynamics and treatment safety

During the bridging period, a significant reduction was observed in both FT3 (mean ΔFT3: -6.2 ± 8.5 pmol/L; median reduction: 27.8%) and FT4 (mean ΔFT4: -15.4 ± 16.1 pmol/L; median reduction: 25.7%) levels (both *P* < 0.001). The mean pre RAI FT3 level in the lithium group was numerically lower than in the standard care group (16.1 vs. 18.9 pmol/L, *P* = 0.065) (**Table 3**).

**Table 3:** Thyroid hormone dynamics and treatment-related events.

Parameter	Lithium-bridging group (n=46)	Standard-care group (n=100)	*P*-value
Hormone reduction (Δ, Lithium only)			
Initial FT3 (post-ATD), pmol/L	22.3 ± 10.1	N/A	–
Initial FT4 (post-ATD), pmol/L	59.9 ± 20.5	N/A	–
ΔFT3 (pmol/L)	-6.2 ± 8.5	N/A	<0.001*
ΔFT4 (pmol/L)	-15.4 ± 16.1	N/A	<0.001*
Pre-RAI hormone level			
FT3 (pmol/L)	16.1 ± 9.3	18.9 ± 8.5	0.065
FT4 (pmol/L)	44.5 ± 18.3	46.8 ± 11.3	0.355
Treatment-related events, n (%)			
Lithium-related GI symptoms	4 (8.7)	0 (0)	0.010†
Post-RAI Thyroid Storm	0 (0)	3 (3.0)	0.559†

*Data are presented as mean ± standard deviation or n (%). GI, gastrointestinal. Within-group comparisons for Δ values used the paired t-test. †P-value derived from Fisher’s exact test.

Lithium was well tolerated. Mild, transient gastrointestinal symptoms were reported in 4 patients (8.7%). No cases of lithium toxicity or significant renal dysfunction were recorded. Serum lithium levels were monitored weekly and maintained within 0.6–1.0 mmol/L; renal function was checked at baseline and before RAI administration, with no significant impairment observed. Neurological symptoms (tremor, polyuria) were actively queried during clinical visits; none were documented. The median duration of lithium therapy was 12 days (IQR 10–14 days). No patient in the lithium bridging group developed thyroid storm (0/46), compared to 3 cases (3.0%) in the standard care group (*P* = 0.559).

### 3.4 Independent predictors in the entire cohort

Multivariate logistic regression analysis of the entire cohort (Model A, n=146) identified pre RAI FT3 as an independent risk factor for treatment failure (adjusted odds ratio [aOR] 1.55 per 5 pmol/L increase, 95% CI 1.15–2.09, *P* = 0.004). Assignment to the lithium bridging group showed a non-significant protective trend (aOR 0.65, 95% CI 0.23–1.85, *P* = 0.420). The Hosmer–Lemeshow test indicated good model fit (P = 0.432). VIF values for all predictors were <3, indicating no significant multicollinearity. Full model details are provided in **Supplementary Table 1**. The full model results are summarized in **Table 4**.

**Table 4:** Factors associated with treatment failure in the full cohort (Model A, n=146).

Variable	Adjusted OR (95% CI)	*P*-value
Lithium Bridging Therapy (Yes vs. No)	0.65 (0.23 – 1.85)	0.420
Pre-Radioiodine FT3 (per 5 pmol/L increase)	1.55 (1.15 – 2.09)	0.004
Pre-Radioiodine FT4 (per 10 pmol/L increase)	1.08 (0.85 – 1.36)	0.536
Age (per 10-year increase)	0.91 (0.67 – 1.24)	0.556
Sex (Female vs. Male)	1.21 (0.49 – 3.00)	0.681
Thyroid Weight (per 10 g increase)	1.12 (0.89 – 1.41)	0.334
Large Goiter (>50g, Yes vs. No)	1.45 (0.52 – 4.02)	0.478

Model diagnostics: Hosmer–Lemeshow P = 0.432, Nagelkerke R² = 0.218, AUC = 0.74 (95% CI 0.63–0.85). VIF values <3 for all predictors. Full model diagnostics are provided in **Supplementary Table 1**.

### 3.5 Predictors of treatment response within the lithium subgroup

Analysis within the lithium treated patients (Model B, n=46) showed that the magnitude of FT3 reduction (ΔFT3) was independently associated with a lower odds of treatment failure (aOR 0.45 per 5 pmol/L decrease, 95% CI 0.22–0.92, *P* = 0.028). Even after accounting for this reduction, the final pre RAI FT3 level remained a significant independent risk factor (aOR 2.10 per 5 pmol/L increase, 95% CI 1.18–3.74, *P* = 0.012). Goodness of fit was satisfactory (*P* = 0.581). Full model details are available in **Supplementary Table 2**, and the key results are presented in **Table 5**.

**Table 5:** Factors associated with treatment failure in the lithium-bridging subgroup (Model B, n=46).

Variable	Adjusted OR (95% CI)	*P*-value
ΔFT3 (per 5 pmol/L decrease)	0.45 (0.22 – 0.92)	0.028
Pre-Radioiodine FT3 (per 5 pmol/L increase)	2.10 (1.18 – 3.74)	0.012
Age (per 10-year increase)	0.88 (0.58 – 1.35)	0.567
Sex (Female vs. Male)	1.32 (0.42 – 4.15)	0.639
Thyroid Weight (per 10 g increase)	1.18 (0.87 – 1.61)	0.289
Large Goiter (>50g, Yes vs. No)	1.89 (0.51 – 7.02)	0.342

Model diagnostics: Hosmer–Lemeshow P = 0.581, Nagelkerke R² = 0.342, AUC = 0.81 (95% CI 0.67–0.95). VIF values <2.5 for all predictors. Full details are provided in **Supplementary Table 2**.

### 3.6 Secondary outcome

Lithium bridging was also associated with improved efficiency of the therapeutic response. The median time from radioiodine administration to the development of either euthyroidism or hypothyroidism was significantly shorter in the lithium-bridging group compared to the standard-care group (2.0 months, IQR 1.5–3.0 vs. 3.0 months, IQR 2.0–4.5, *P* = 0.032).

## 4 Discussion

### 4.1 Principal findings

This study demonstrates that a short term lithium carbonate bridging strategy may be associated with a different therapeutic profile of radioiodine in patients with severe Graves’ disease. Compared to standard care, this approach was associated with a higher rate of euthyroidism and a lower rate of hypothyroidism at 6 month follow up. This was achieved with a low incidence of mild, manageable side effects and no cases of thyroid storm in the lithium group.

### 4.2 Reaffirming pre radioiodine FT3 as a key modifiable factor

Our findings robustly quantify the central role of pre-ablation thyroid hormone levels in determining therapeutic success. Multivariate analysis confirmed that a higher pre−radioiodine FT3 level is the strongest independent modifiable risk factor for persistent hyperthyroidism. This reinforces the underlying pathophysiology: a gland in a hyperactive, high-FT3 state is characterized by accelerated follicular cell turnover and proliferation, which is associated with reduced sodium−iodide symporter (NIS) expression and functional radioresistance (8, 9), potentially mediated through TSH suppression or other pathways (16). Therefore, actively lowering FT3 prior to therapy may represent a strategic approach to overcome a key biological barrier to cure.

### 4.3 Mechanistic insight: association with FT3 reduction

The most significant contribution of this analysis is the exploration of a direct, quantitative link between lithium’s action and outcomes. Within the lithium treated subgroup, the magnitude of FT3 reduction (ΔFT3) was independently associated with treatment success. This dose response relationship suggests that the degree of FT3 lowering achieved during the bridging period may be an important determinant of outcome. We hypothesize that lithium may exert its effect by pharmacologically “cooling” the hypermetabolic thyroid gland. By inhibiting hormone secretion, it reduces the intrathyroidal thyrotoxic drive, potentially enhancing radioiodine efficacy through dual pathways: (1) Cell cycle modulation: reducing proliferative stimuli may slow follicular cell turnover, potentially increasing the proportion of cells in radiosensitive phases; (2) NIS function: alleviating the hypermetabolic state may improve functional expression or activity of NIS (17, 18), thereby enhancing radioiodine uptake and retention. These mechanistic interpretations, however, remain exploratory and require confirmation in prospective studies.

### 4.4 Interpretation of the outcome pattern

The observation that the lithium group had both a higher euthyroidism rate and a lower hypothyroidism rate compared to the standard care group appears paradoxical at first glance. We propose several potential explanations. First, lithium may exert a dual effect: by reducing pre RAI FT3 levels, it lowers the functional “hormonal load” of the gland, while its property of prolonging intrathyroidal iodine retention may result in a more graded, rather than abrupt, thyroid ablation. This could increase the likelihood of achieving euthyroidism without immediately causing hypothyroidism. Second, the 6 month time point may capture an intermediate state before eventual progression to hypothyroidism. Third, residual confounding or selection bias (e.g., patients with milder disease being selected for lithium) cannot be excluded. These findings should therefore be interpreted as hypothesis generating and warrant further prospective investigation.

### 4.5 Safety

Lithium was well tolerated in this cohort. The low incidence of gastrointestinal side effects (8.7%) and absence of lithium toxicity or renal impairment are consistent with previous reports of short term lithium use (10). The trend toward zero thyroid storm cases in the lithium group, while not statistically definitive, is clinically encouraging given the life threatening nature of this complication (19). By potentially preventing rebound thyrotoxicosis, lithium bridging may mitigate a risk inherent to the standard management approach.

### 4.6 Limitations

This study has several limitations inherent to its retrospective, single center design, which may introduce selection bias and unmeasured confounding despite comparable baseline characteristics on measured variables. The decision to prescribe lithium was physician dependent, and we may not have captured all factors influencing treatment selection (confounding by indication). Although we adjusted for a comprehensive set of covariates, residual confounding may persist. The sample size, while adequate for the primary outcome, provided limited power to detect differences in rare safety events such as thyroid storm. *Post hoc* power calculation indicated that with the observed event rates and sample size, the study had 80% power to detect an odds ratio of approximately 2.5 for the lithium effect, meaning smaller but clinically relevant effects may not have been detected. The lack of systematic data on the exact duration of the ATD free interval or beta blocker dosing in the standard care group is another constraint. Furthermore, the 6 month follow up period captures initial treatment response but not long term stability or relapse rates, which are known to be influenced by ongoing autoimmune activity (20). Finally, while we used FT3 as the primary hormone parameter, total T3 is used in some regions; however, FT3 provides a comparable assessment of thyrotoxicosis severity.

### 4.7 Conclusion and future directions

In conclusion, pre radioiodine lithium carbonate bridging may be associated with improved treatment outcomes in severe Graves’ disease, with a higher likelihood of euthyroidism and a lower rate of hypothyroidism at 6 months. This association may be mediated through reduction in serum FT3 levels, which is a key modifiable predictor of treatment failure. These findings provide a rationale for prospective randomized controlled trials to definitively evaluate the role of lithium bridging in high risk patients undergoing radioiodine therapy.

## Data Availability

The original contributions presented in the study are included in the article/**Supplementary Material**. Further inquiries can be directed to the corresponding author.
