# Supplementary material for: Lithium carbonate bridging and outcomes of radioiodine therapy in severe Graves’ disease: a retrospective cohort study
Source: Front Endocrinol (Lausanne). 2026 May 7;17:1770772. doi: 10.3389/fendo.2026.1770772 (PMC13189748; doi:10.3389/fendo.2026.1770772)
Supplement: Supplementary file 1 [file Table1.docx]

Supplementary Material

# Supplementary Tables

## Supplementary Table S1

**Table S1: Full Multivariate Logistic Regression Model for the Entire Cohort (Model A)**

| Variable | Adjusted OR (95% CI) | *P*-value |
| --- | --- | --- |
| Lithium Bridging Therapy (Yes vs. No) | 0.65 (0.23 – 1.85) | 0.420 |
| Pre‑Radioiodine FT3 (per 5 pmol/L increase) | 1.55 (1.15 – 2.09) | 0.004* |
| Pre‑Radioiodine FT4 (per 10 pmol/L increase) | 1.08 (0.85 – 1.36) | 0.536 |
| Age (per 10-year increase) | 0.91 (0.67 – 1.24) | 0.556 |
| Sex (Female vs. Male) | 1.21 (0.49 – 3.00) | 0.681 |
| Thyroid Weight (per 10 g increase) | 1.12 (0.89 – 1.41) | 0.334 |
| Large Goiter (>50g, Yes vs. No) | 1.45 (0.52 – 4.02) | 0.478 |

**Model Diagnostics:**

Hosmer–Lemeshow goodness‑of‑fit: χ² = 7.32, *P* = 0.432

Nagelkerke *R*² = 0.218

Area under ROC curve (AUC) = 0.74 (95% CI 0.63–0.85)

VIF values for all predictors < 3.0, indicating no significant multicollinearity

## Supplementary Table S2

**Table S2: Full Multivariate Logistic Regression Model for Lithium-Bridging Group Only (Model B)**

| Variable | Adjusted OR (95% CI) | *P*-value |
| --- | --- | --- |
| ΔFT3 (per 5 pmol/L decrease) | 0.45 (0.22 – 0.92) | 0.028* |
| Pre‑Radioiodine FT3 (per 5 pmol/L increase) | 2.10 (1.18 – 3.74) | 0.012* |
| Age (per 10-year increase) | 0.88 (0.58 – 1.35) | 0.567 |
| Sex (Female vs. Male) | 1.32 (0.42 – 4.15) | 0.639 |
| Thyroid Weight (per 10 g increase) | 1.18 (0.87 – 1.61) | 0.289 |
| Large Goiter (>50g, Yes vs. No) | 1.89 (0.51 – 7.02) | 0.342 |

Model Diagnostics:

Hosmer–Lemeshow goodness‑of‑fit: χ² = 5.82, *P* = 0.581

Nagelkerke *R*² = 0.342

Area under ROC curve (AUC) = 0.81 (95% CI 0.67–0.95)

VIF values for all predictors < 2.5, indicating no significant multicollinearity

## Supplementary Table S3

**Table S3: Sensitivity Analysis – FT3 as Continuous Variable (per 1 pmol/L Increment/Decrement)**

**A. Full Cohort Analysis (n=146)**

| Variable | Adjusted OR (95% CI) | *P*-value |
| --- | --- | --- |
| Lithium Bridging Therapy (Yes vs. No) | 0.63 (0.21 – 1.88) | 0.402 |
| Pre‑Radioiodine FT3 (per 1 pmol/L increase) | 1.09 (1.04 – 1.15) | 0.001* |
| Pre‑Radioiodine FT4 (per 10 pmol/L increase) | 1.07 (0.84 – 1.36) | 0.588 |
| Age (per 10-year increase) | 0.92 (0.68 – 1.25) | 0.595 |
| Sex (Female vs. Male) | 1.18 (0.47 – 2.96) | 0.724 |
| Thyroid Weight (per 10 g increase) | 1.10 (0.87 – 1.39) | 0.421 |
| Large Goiter (>50g, Yes vs. No) | 1.42 (0.51 – 3.96) | 0.501 |

**B. Subgroup Analysis – Lithium Group Only (n=46)**

| Variable | Adjusted OR (95% CI) | *P*-value |
| --- | --- | --- |
| ΔFT3 (per 1 pmol/L decrease) | 0.90 (0.82 – 0.99) | 0.031* |
| Pre‑Radioiodine FT3 (per 1 pmol/L increase) | 1.14 (1.05 – 1.24) | 0.003* |
| Age (per 10-year increase) | 0.89 (0.59 – 1.34) | 0.582 |
| Sex (Female vs. Male) | 1.28 (0.41 – 4.02) | 0.671 |
| Thyroid Weight (per 10 g increase) | 1.16 (0.85 – 1.58) | 0.341 |
| Large Goiter (>50g, Yes vs. No) | 1.82 (0.49 – 6.76) | 0.372 |

Interpretation: Results from this sensitivity analysis, using FT3 as a continuous variable (per 1 pmol/L increment/decrement), are consistent with the primary analysis using 5 pmol/L increments. Pre‑RAI FT3 remains a significant risk factor for treatment failure, and ΔFT3 remains a significant protective factor in the lithium subgroup. These findings confirm the robustness of the primary analysis and support the clinical relevance of the chosen 5 pmol/L increment.
